# A portfolio of geographically distinct laboratory-adapted *Plasmodium falciparum* clones with consistent infection rates in *Anopheles* mosquitoes

**DOI:** 10.1186/s12936-021-03912-x

**Published:** 2021-09-26

**Authors:** Marga van de Vegte-Bolmer, Wouter Graumans, Rianne Stoter, Geert-Jan van Gemert, Robert Sauerwein, Katharine A. Collins, Teun Bousema

**Affiliations:** 1 Radboud Institute for Health Sciences and Radboud Center for Infectious Diseases, Radboud University Medical Center, Nijmegen, The Netherlands; 2 Department of Immunology and Infection, London School of Hygiene and Tropical Medicine, London, UK; 3 TropIQ Health Sciences, Nijmegen, The Netherlands

**Keywords:** Malaria, Transmission, Parasite culture, Culture adaptation, Cloning, Clinical isolates, *Anopheles*, Gametocyte, Oocysts

## Abstract

**Background:**

The ability to culture *Plasmodium falciparum* continuously in vitro has enabled stable access to asexual and sexual parasites for malaria research. The portfolio of isolates has remained limited and research is still largely based on NF54 and its derived clone 3D7. Since 1978, isolates were collected and cryopreserved at Radboudumc from patients presenting at the hospital. Here, procedures are described for culture adaptation of asexual parasites, cloning and production of sexual stage parasites responsible for transmission (gametocytes) and production of oocysts in *Anopheles* mosquitoes. This study aimed to identify new culture-adapted transmissible *P. falciparum* isolates, originating from distinct geographical locations.

**Methods:**

Out of a collection of 121 *P. falciparum* isolates stored in liquid nitrogen, 21 from different geographical origin were selected for initial testing. Isolates were evaluated for their ability to be asexually cultured in vitro, their gametocyte production capacity, and consistent generation of oocysts.

**Results:**

Out of 21 isolates tested, twelve were excluded from further analysis due to lack of mature gametocyte production (n = 1) or generation of satisfactory numbers of oocysts in mosquitoes (n = 11). Nine isolates fulfilled selection criteria and were cloned by limiting dilution and retested. After cloning, one isolate was excluded for not showing transmission. The remaining eight isolates transmitted to *Anopheles stephensi* or *Anopheles coluzzii* mosquitoes and were categorized into two groups with a reproducible mean oocyst infection intensity above (n = 5) or below five (n = 3).

**Conclusions:**

These new *P. falciparum* culture-adapted isolates with reproducible transmission to *Anopheles* mosquitoes are a valuable addition to the malaria research tool box. They can aid in the development of malaria interventions and will be particularly useful for those studying malaria transmission.

**Supplementary Information:**

The online version contains supplementary material available at 10.1186/s12936-021-03912-x.

## Background

In 1912, Bass and Johns described successful culture of *Plasmodium* asexually in vitro [1]; in 1976, Trager and Jensen achieved continuous in vitro cultivation of *Plasmodium falciparum* [2, 3], providing for the first time stable access to large quantities of stage specific malaria parasites [2], including sexual stages, the so-called gametocytes [4]. In search of an isolate for stable continuous in vitro culture and transmission experiments, the Nijmegen falciparum strain 54 (NF54) was selected. This was a case of airport malaria in a patient residing near Schiphol international airport in the Netherlands in 1979 [5]. This isolate, thought to have originated from West-Africa [6], and its clone 3D7 [7] are arguably the most widely used laboratory strains of *P. falciparum* and have become a standard resource for malaria research. They have formed the basis of whole sporozoite vaccination approaches [8], and the basis of many functional assays for evaluation of malaria interventions, such as the standard membrane feeding assay (SMFA) [9]. Given the importance of genetic variation for vaccine and drug efficacy, reliance on a single parasite isolate fails to give a comprehensive insight into intervention potency in natural infections [10]. Similarly, relevant inter-strain variation in parasite growth rates [11], gametocyte production [12] and sporozoite invasion capacity [13, 14] warrant further examination. The current portfolio of laboratory isolates for studies on sexual and sporogonic stages is very limited. During continuous culture, isolates can acquire spontaneous mutations and lose their ability to sexually differentiate [15], hampering utility of many *Plasmodium* isolates for malaria research. Expanding the portfolio to include a diversity of isolates would be beneficial. This study describes selection, clonal adaptation, and evaluation of *P. falciparum* isolates for in vitro culture and sporogonic stage development.

## Methods

### Reagents and in vitro culture conditions

#### RPMI culture medium

0.5 g of hypoxanthine (Sigma, art nr. H-9377-25G) was added to 2 l Milli-Q and placed on a magnetic stirring plate at room temperature (RT) to dissolve. Subsequently 59.4 g HEPES (BDH Prolabo, 441487M or alternative 441476L) and 1 pot RPMI1640 powder (Life Technology Invitrogen, 518-00035) was added. The volume was topped up with Milli-Q to 4800 ml and left to dissolve by magnetic stirring. Media was transferred to a pressure vessel and an additional 4800 ml Milli-Q was added, giving a total volume of 9600 ml. The pressure vessel inlet was attached to a N_2_ gas cylinder and the outlet to silicon tubing with a 0.2 µm hollow fiber medium filter (Mediakap-10 Spectrum, me2m-10b-12s). Under 1 bar of pressure, the media was filter sterilized and collected in a customized sterile 3000 ml Erlenmeyer with tap placed inside a sterile biological safety cabinet. Media was filled out in 80 ml aliquots in sterilized glass bottles that were closed with aluminum caps with rubber inlay and frozen at − 20 °C. Medium was shortly placed in a water bath to thaw at 37 °C before use. For so-called incomplete medium, 3.7 ml of autoclaved 5% sodium hydrogen bicarbonate (Merck, 1.06329.1000) in Milli-Q was added. For complete medium, another 8 ml pooled human serum was supplemented. Thawed medium was stored in the fridge at 4 °C and used for a maximum of 7 days.

#### Serum

Serum was obtained in 200 ml IV bags by the national blood bank (Sanquin; Nijmegen, The Netherlands). Bags of at least 10 malaria-naïve donors (group A or AB, Rh + and Rh− mixed) were pooled and collected in a customized 3000 ml Erlenmeyer with tap. Aliquots of 100 ml were filled out in sterilized glass bottles and closed with aluminum caps with rubber inlay, frozen at − 20 °C and thawed in a water bath at 37 °C before use. Thawed serum was stored in the fridge at 4 °C and used for a maximum of 7 days.

#### Erythrocytes

Blood from malaria naïve donors (group O, Rh+ and Rh− blood mixed) was drawn twice weekly in 10 ml lithium heparinized tubes (BD, Vacutainer) after skin disinfection with 70% Isopropyl Alcohol (FA Alcohol Swab-S), by the national blood bank (Sanquin; Nijmegen, The Netherlands). Tubes were centrifuged at 750×*g* for 5 min in a benchtop centrifuge at RT. After removal of the plasma and buffy-coat layer, blood from at least 6 donors was pooled and transferred to 15 ml tubes (Corning, 430766). Blood was washed 2 × by adding 10 ml incomplete medium to each tube and centrifuged as above. After removal of the supernatant the volume of erythrocytes was diluted with an equivalent volume of complete medium to obtain a 50% haematocrit solution. Tubes were stored in the fridge at 4 °C and used for a maximum of 7 days for parasite culture or 9 days for SMFA blood meal preparation. Unprocessed heparin blood was used for routine mosquito colony maintenance.

#### Culture conditions

Parasites were cultured at 37 °C and continually supplied with a pre-mixed gas mixture of 3% O_2_, 4% CO_2_ and 93% N_2._ Isolates were adapted in an semi-automated shaker system [16], a modified version of the 1981 Butcher model [17], and cultured for gametocyte production in a semi-automated tipper system [18].

### Plasmodium falciparum collection and culture adaptation

Peripheral blood was collected in EDTA Vacutainers (BD). Tubes were spun down for 5 min at 750×*g* at RT in a tabletop centrifuge without break. The plasma and buffy-coat layer was removed before erythrocytes were transferred to a 15 ml tube (Corning, 430766). Subsequently, blood was washed 2 × by topping up the volume with incomplete RPMI medium. Tubes were gently mixed by inverting and spun down as above. After the final wash the supernatant was removed and a Giemsa-stained blood smear was made from the pellet to assess parasites morphologically and counting the percentage of infected erythrocytes. 0.2 ml of patients’ blood was added to 0.1 ml of 50% pre-washed erythrocytes (as described above) and topped up to 10 ml with complete RPMI medium, supplemented with AB serum. Cultures were transferred to the semi-automated shaker system [16]. After one week adaptation serum type A was used for culture. Every 2–3 days parasitaemia was evaluated by microscopy after Giemsa staining and 0.5–1.0% erythrocytes were added until parasites were adapted and 5% haematocrit was reached.

### Cryopreservation and retrieval of parasites

For cryopreservation, a modified methodology of Diggs et al*.* was used [19]. Briefly, cultures were transferred to a 15 ml tube (Corning, 430766) and spun down at 750×*g* for 5 min in a benchtop centrifuge at RT. The supernatant was removed and the residual volume of the erythrocyte pellet was determined. 1 × the volume of complete culture medium was added to obtain 50% haematocrit. Subsequently 2 × the initial volume of 30% glycerol in PBS (Gibco 10010-015) was added dropwise and the volume was gently mixed with a pipette. A minimum of 0.5 ml of suspension was distributed per cryovial (Nunc, 368632). Vials were transferred to a ‘Mr frosty’ freezing container (Nalgene, 5100-0001) and frozen at – 80 ºC overnight before they were placed in liquid nitrogen at – 196 ºC for long term storage.

To retrieve isolates from liquid nitrogen a modified methodology of Diggs et al. was used [19]. Briefly, parasites were retrieved from liquid nitrogen by rapidly thawing of the suspension at 37 °C. After thawing the vial was directly placed on ice. The volume was determined and transferred to a 15 ml tube (Corning, 430766). Two volumes of 27% sorbitol (Merck, 1.07758) in 1 × PBS (Gibco 10010-015) was dropwise added via the side of the inclined tube. The tube was gently mixed by rotating in a nearly horizontal position before it was placed on ice for 12 min. Thereafter, two volumes of cold 5% sorbitol in 1 × PBS was added and the tube was again gently mixed before incubated on ice for 10 min. The tube was centrifuged at 300×*g* for 5 min in a benchtop centrifuge set at 4 °C. The supernatant was removed and the pellet was suspended in 2 volumes of 5% sorbitol and incubated on ice for 8 min. After centrifugation at 750×*g* for 5 min the supernatant was removed and the pellet was washed 2 × with 10 ml incomplete culture medium. After the final wash, 10 ml complete medium and 2.5% fresh erythrocytes were added. Every 2–3 days, growth was assessed after Giemsa staining and 1% of erythrocytes was added, based on parasitaemia, until 5% haematocrit was reached.

### Plasmodium falciparum routine asexual- and sexual parasite culture

To maintain asexual parasite growth, cultures were Giemsa-stained two (tipper system [18]) or three times (shaker system [16]) a week, irrespective of the parasitaemia, to assess the percentage of infected erythrocytes by microscopy at 1000 × magnification. Cultures were diluted back to 0.5 or 1.0% parasitaemia, for respectively shaker or tipper system, and 5% haematocrit per 10 ml by adding erythrocytes. Successful culture adaptation of isolates was determined as continued asexual growth with increasing parasitaemia after cyclic replication.

For gametocyte production, 1% parasitaemia and 5% haematocrit was seeded at day zero in the tipper system [18]. Successful gametocyte culture was evaluated by assessing induction, maturation and transmission. Gametocyte induction and maturation were assessed microscopically eight days after seeding by the presence of stage II gametocytes (D-shaped), and fourteen days after seeding by the presence of mature stage V male and female gametocytes, using the nomenclature classification of Hawking et al. [20] as demonstrated in Ponnudurai et al*.* [16]. Special attention was paid to; (a) thickness of the gametocyte, (b) pace of development, and (c) localization of the pigment. Mature male gametocytes were activated to test for their ability to exflagellate. To assess exflagellation, evaluated 14 days post seeding, 200 µl of culture material was spun down with a benchtop centrifuge at 17,700×*g* for 20 s. After removal of the supernatant, 3 µl of pelleted erythrocytes was mixed on a microscope slide with 10 µl of Fetal Bovine Serum (FBS, Invitrogen, art no 10270106) and incubated for 10 min at room temperature in a humidified box. A coverslip with Vaseline coated edges was applied on top and exflagellation was assessed by microscopy at 400 × magnification and determined as the presence or absence of exflagellation centres without further quantification.

Cultures with the demonstrated ability to exflagellate were fed to mosquitoes to test their ability to establish infection [21]. An infective blood meal was prepared as previously described [21]. In brief at 37 °C, 300 µl of culture material was added to 180 µl pellet erythrocytes and spun down in a benchtop centrifuge as described above. Supernatant was removed, without disturbing the pellets surface, and 150 µl serum was added. Blood meal was gently vortexed and fed by midi-feeders to a total of 50 mosquitoes.

### Cloning of isolates

For cloning, a modified methodology of Thaithong was used [22]. Briefly, a 100 × dilution of culture material was prepared to count the number of erythrocytes with a haemocytometer. The number of infected erythrocytes per µl was calculated. Three dilutions were made in complete medium with 1% fresh erythrocytes; 100, 10 and 0.3 parasites per 100 µl. For each dilution 100 µl was transferred to respectively 10, 10 and 40 wells of a 96 well flat-bottom microplate (Nunc, 167008), outer wells were filled with 150 µl sterile MilliQ to diminish evaporation. Plates were maintained in humidified modular chamber (Billups and Rothenberg) at 37 °C and an atmosphere of 3% O_2_, 4% CO_2_, and 93% N_2_. Medium was changed every other day, by placing the plate on a 45 degrees angle stand for 30 min before removing the medium carefully without disturbing the settled erythrocytes at the bottom of the wells. Every other day of medium change 0.5% fresh erythrocytes were added. After 10 days, thick smears were prepared of the control wells, that were seeded with 10 and 100 parasites. Without fixation they were Giemsa-stained to confirm parasite growth. After two to three weeks 10 µl of each experimental well was analyzed for growth by microscopy after Giemsa staining. For positive wells, usually maximal 10 per plate, the remaining culture volume was transferred to a shaker flask and 1% fresh erythrocytes were added. Every 2–3 days growth was assessed by microscopy and ~ 0.5% of fresh erythrocytes was added, based on parasiaemia, until 5% haematocrit was reached.

Clonality was confirmed by assessment of the merozoite surface protein 1 and 2 (MSP1 and MSP2) and Glutamate-Rich Protein (GLURP) polymorphic region by PCR, as described before [14], a methodology adapted from Snounou et al*.* [23]. DNA was extracted from pelleted cultures using the QIAamp DNA Blood Kit spin protocol for DNA purification from blood or body fluids (Qiagen, 51104). GoTaq G2 Flexi DNA Polymerase kit (Promega, M7801) was used for DNA amplification. Only if single bands were observed for each of the three target genes, clonality was assumed.

### Mosquito husbandry and infection

*Anopheles stephensi* Sind-Kasur strain [24] and *Anopheles coluzzii* Ngousso strain [25], were reared at 30 °C and ~ 80% humidity, with a 12 h reverse day/night cycle. Culture material was offered to 1–5 days old female mosquitoes using a glass membrane midi feeder system [21]. Unfed and partially fed mosquitoes were removed after feeding and mosquitoes were maintained on 5% glucose at 26 °C and ~ 80% humidity. Transmission was assessed 6–8 days after feeding by counting the number of oocysts on mosquito midguts after staining with 1% mercurochrome [21].

## Results

### Collection and selection of patient isolates

**Fig. 1:**
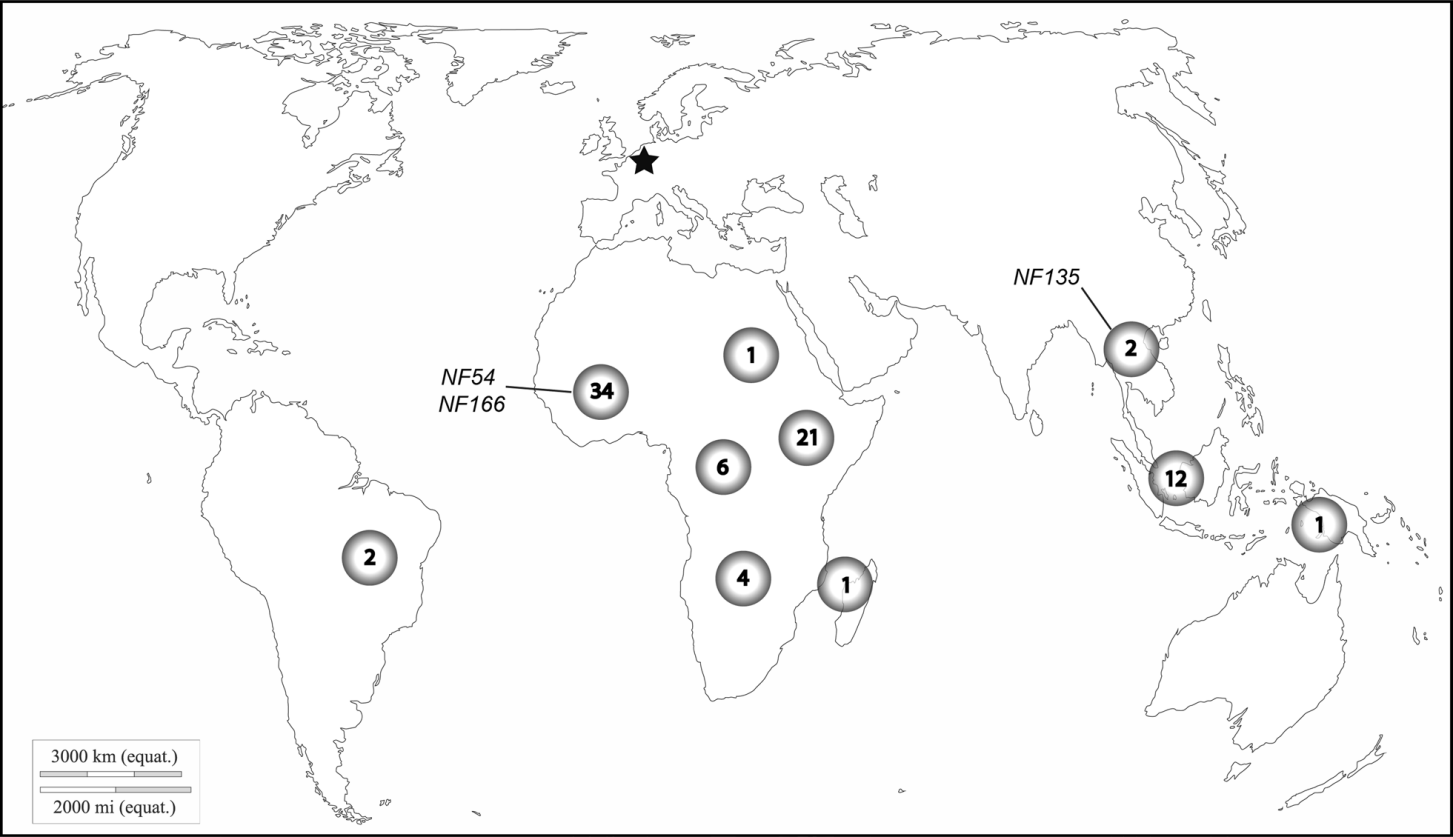
Origin of collected *Plasmodium falciparum* isolates. This world map presents the number of isolates and their approximate origin (d-maps.com^©^). From 1978 onwards 121 isolates derived from patients with clinical symptoms were collected by the Radboudumc (depicted by asterisk) and stored in liquid nitrogen. The origin of three isolates, previously published by the Radboudumc, is shown in text (NF54, NF135 and NF166). For 32 collected isolates the origin was unknown and for 5 isolates uncertain (not shown in figure)

Since 1978, *P. falciparum* isolates derived from various geographical locations were collected from patients prior to anti-malarial drug treatment at the Radboudumc and other hospitals in the Netherlands. Over a period of 40 years, 121 isolates were stored in liquid nitrogen. The majority of isolates originated from the African continent. The origin of 32 isolates was unknown and for 5 isolates uncertain. Three have already been reported; NF54 (West-African) [26], NF135 (Cambodia) [27] and NF166 (Guinea) [14]. Aiming for variation in terms of origin, 21 isolates were selected for initial testing. These samples were collected between 1990 and 2019 from malaria patients that visited countries in the following continents; South America (1), Central Africa (2), West Africa (9), East Africa (3), Southeast Africa (1), South Asia (1), Southeast Asia (3) and Oceania (1) (Fig. 1; Additional file 2: Table S1).

### Assessment of selected isolates

**Fig. 2:**
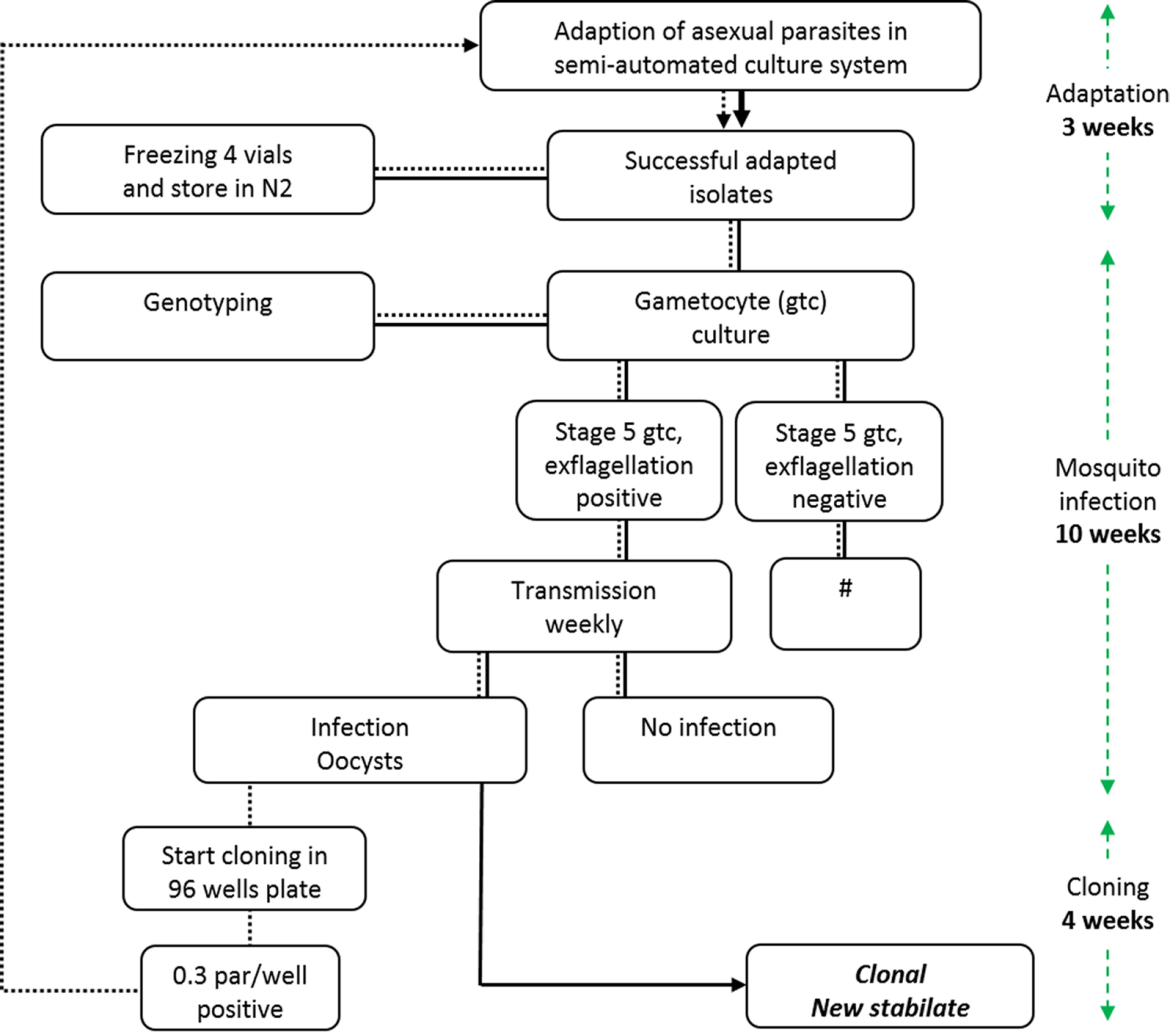
Screening *Plasmodium falciparum* patient isolates. Flow diagram of screening and selection procedure. Vials from liquid nitrogen were thawed and isolates were tested for adaptation to the automated shaker culture system (dashed line) [16]. After adaptation vials were frozen to expand the number of aliquots. Isolates were genotyped, transferred to automated tipper culture system [18] and tested for their ability to produce viable gametocytes that can initiate transmission to mosquito vector. Successful isolates were cloned by limiting dilution, cryopreserved and tested again after thawing (solid line). The minimum time from adaptation to successful cloning and establishment of a new clonal isolate was estimated 30 weeks

The 21 selected isolates were evaluated for their suitability for (i) in vitro culture, (ii) gametocyte production, and (iii) transmission to mosquitoes. The evaluation was performed as detailed in the flow diagram in Fig. 2. Isolate transmission was evaluated in *An. stephensi* mosquitoes, with the exception of NF165 that was also evaluated in *An. coluzzii* in a different study [28].

Patient isolates were retrieved from liquid nitrogen, cultured in the shaker system, a system designed for optimal culture conditions [16]. Parasite growth was monitored every two to three days by Giemsa-stained blood smears. When parasitaemia reached 5% rings, after a maximum of 3–4 weeks, new aliquots were frozen to expand the number of available vials. Subsequently the culture was seeded to assess the ability to produce gametocytes, from which later a sample was withdrawn for genotyping. All 21 selected isolates were successfully adapted to asexual in vitro culture conditions.

Following adaptation, isolates were transferred to the tipper system for static culture conditions, favorable for gametocyte transmission [18]. A drop in parasite growth was consistently observed the first days after transfer, sometimes accompanied by a temporary increase in gametocytaemia. After adaptation to static conditions, cultures were regularly diluted with erythrocytes to maintain asexual growth or seeded for gametocyte production. A common characteristic for gametocyte producing isolates was an observed slow asexual multiplication that prevented the culture from overgrowing and crashing before gametogenesis was initiated. Gametocytes induced in the first cycles after initiating in vitro culture from liquid nitrogen were often found to not transmit to mosquitoes during that early stage.

**Fig. 3:**
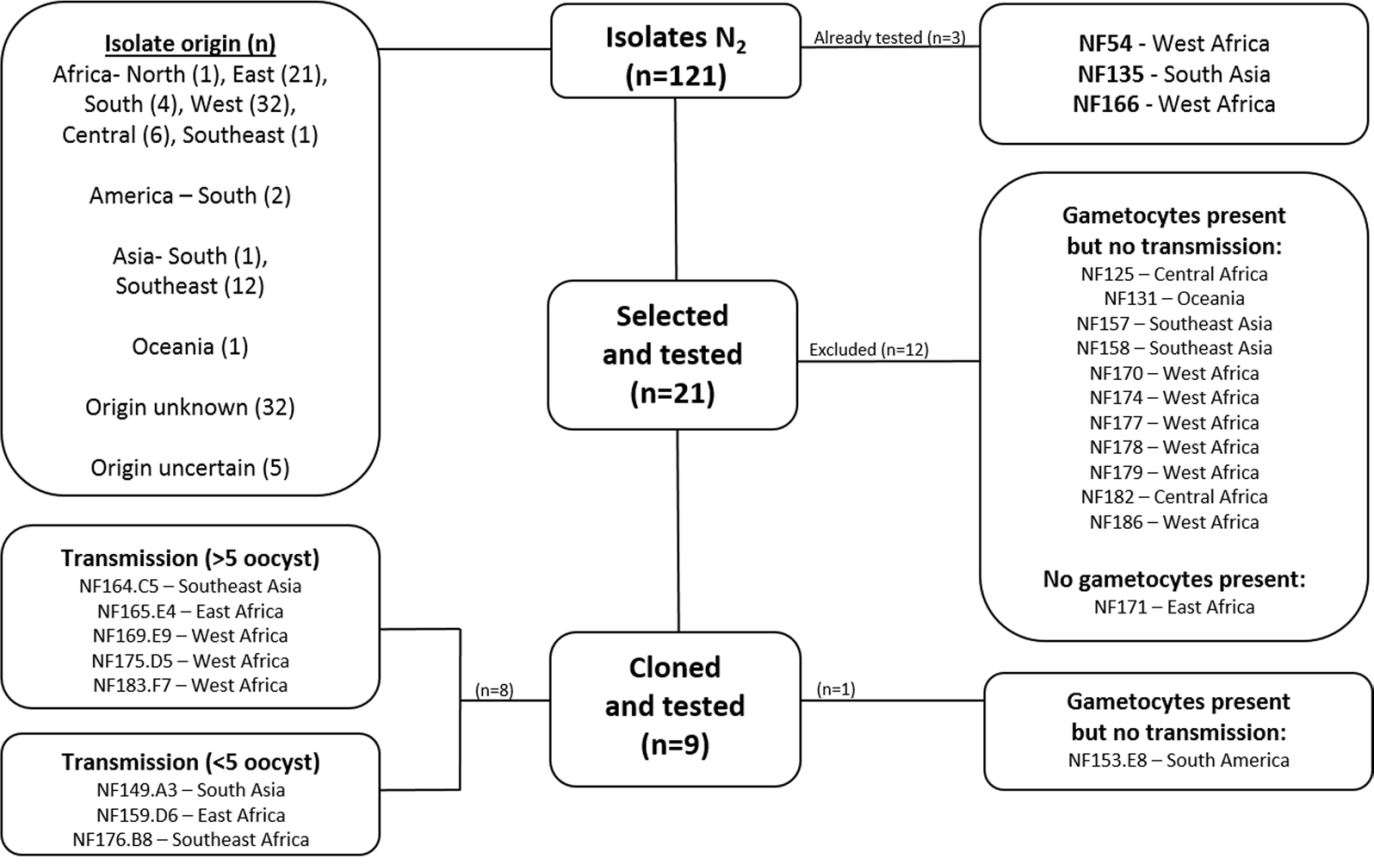
Selection and exclusion of *Plasmodium falciparum* isolates. Flow diagram of in- and excluded isolates in this study. Out of 121 isolates 21 were selected on their origin and tested according to the process presented in Fig. 2. Out of this selection 12 were excluded for cloning: 11 isolates showed no transmission and 1 produced no gametocytes. The remaining 9 isolates were cloned by limiting dilution and tested subsequently. After cloning, 1 isolate was excluded for showing no transmission (NF153.E8) and 8 isolates were found eligible for use in future transmission research. Eligible isolates were categorized on transmission intensity into two groups with mean infection intensities above or below 5 oocysts per mosquito

Out of 21 isolates, one failed to produce gametocytes and was not examined further. Eleven isolates produced gametocytes but showed no transmission in the membrane feeding assays (i.e. no oocysts observed) and were not taken forward. For one of these isolates that failed to establish mosquito infection gametes/zygotes were observed 24 h post feeding that did not develop into ookinetes (NF125). The remaining 9 isolates were found eligible for further analysis after passing the selection criteria; in vitro culture adaptation, gametocyte production, and mosquito transmission (Fig. 3).

**Fig. 4:**
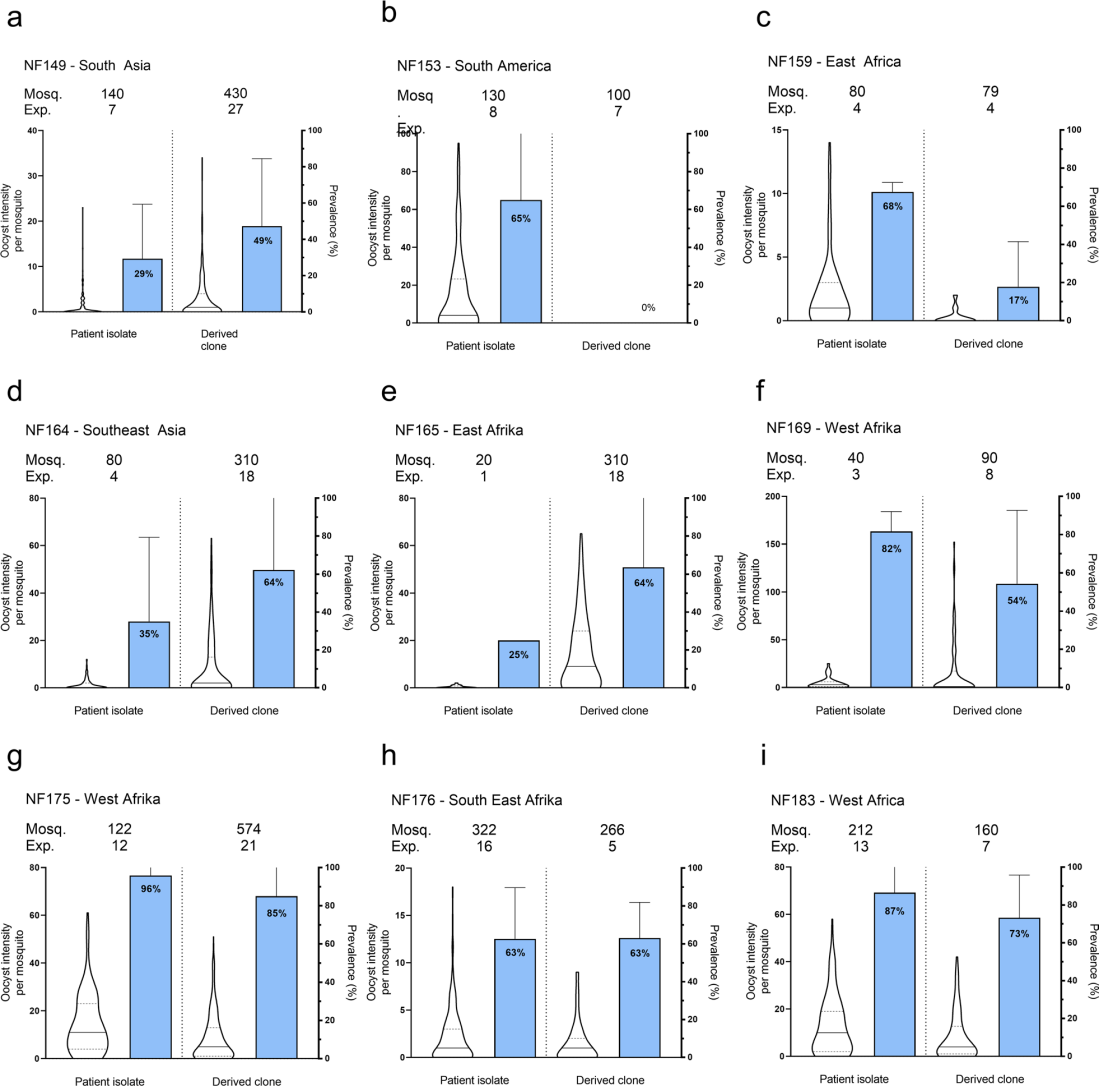
Transmission intensity and infection prevalence of nine patient isolates and their derived clones. Violin plots present infection intensity, the number of established oocysts on the mosquito midgut post infection, with lines indicating the median and quartiles. Bar columns show infection prevalence (the percentage of infected mosquitoes) with SD. The number of mosquitoes (mosq.) indicates number of dissected midguts in individual experiments (exp.). Clonal isolates were categorized on intensity, some clones repeatedly achieved mosquito infection intensities with a mean oocyst density above 5 (panel **d**, **f**, **g** and **i**) whilst others below 5 (panel **a**, **c** and **h**). Gametocytes of one isolate lost the ability to transmit to mosquito vector after cloning (panel **b**)

To generate clonal lines, cultures were initiated from ring stage parasites. This was achieved by using freshly thawed vials, since only rings survive the freeze–thaw process. Nine isolates were used for cloning by limiting dilution when parasitaemia reached 5%. Clonal isolates were tested again for the three selection criteria (Fig. 2). All clonal isolates showed asexual parasite replication with formation of gametocytes; one isolate lost the ability to establish mosquito infection (NF153.E8) due to poor maturation of gametocytes observed microscopically. In total, eight clonal isolates retained infectivity to mosquitoes (Fig. 3); six of these were from Africa and two from Asia. Infection prevalence and intensity are plotted for the original patient isolate and their derived clone (Fig. 4). Although the number of replicates is too small to conclude consistency in infection intensity, some clones repeatedly achieved mosquito infection intensities with a mean oocyst density above 5 (NF164.C5. NF165.E4, NF169.E9, NF175.D5 and NF183.F7) whilst others achieved mosquito infection intensities with a mean oocyst density below 5 (NF149.A3, NF159.D6 and NF176.B8) (Additional file 2: Table S2).

## Discussion

This study aimed to identify new transmissible *P. falciparum* isolates. Isolates were selected based on ability to be cultured in vitro, produce gametocytes and transmit reproducibly to mosquitoes. The current approach to expand the portfolio of parasite isolates for transmission did not exhaustively test individual isolates but aimed to select isolates most readily adaptable to gametocyte culture. Thus, isolates that did not directly fulfill quality criteria after initial culture were rapidly disqualified. The presented effort to identify new gametocyte-producing lines highlighted that slow asexual multiplication was favourable, preventing the culture from overgrowing and crashing before gametogenesis was initiated. It is worth noting that gametocytes induced shortly after in vitro culture initiation from liquid nitrogen were often not transmissible to mosquitoes during that early phase. Equally important, isolates maintained in culture for long periods can lose the ability to produce gametocytes, as described in a review by Ngotho and colleagues [29]. Therefore, in order to have stable transmission cryopreservation of isolates and maintaining a culture for a short period, typically 3 months, is very important. The presence of morphologically matured gametocytes [16, 20] in cultures, and their quantity, did not always reflect transmission potential in the current study, as apparent for isolate NF153.E8. Thus, stable gametocyte production does not always equal stable transmission. Transmission to mosquitoes may fluctuate over time and even in a highly controlled laboratory environment with standardized protocols transmission remains challenging and to some extent unpredictable. This is illustrated by mosquito infection intensity over time for NF54 and NF175.D5 (Additional file 1: Fig. S1). Between identical vials retrieved from liquid nitrogen, there can be substantial variation in the performance of the isolate. These unexplained variations in infectivity where beyond the scope of this study, but are most likely due biological variation e.g. gametocyte quality and mosquito susceptibility. Of the selected clones, each vial consistently produced infectious gametocytes. Whilst interesting variation in infection intensity in mosquitoes was observed between clones, further research is warranted before conclusions can be drawn on the stability of these apparent transmission phenotypes.

This study used specialized semi-automated culture systems. Shaker systems are optimized for asexual parasite growth reaching high parasitaemia due to optimal gas exchange, access to nutrition, and efficient merozoite invasion. In contrast, static tipper systems are designed to produce fertile sexual stages suitable for mosquito infection [18]. Adaptation of presented isolates to alternative systems and culture conditions will require parasite adaptation, it is unknown if this can result in a reduction or loss of parasite growth or transmission. A limitation of the current work is that isolates were only tested in *An. stephensi* [24], with exception of NF165 that was also tested in *An. coluzzii* as part of another study [28]. *Anopheles stephensi* is an important vector for malaria in urban environments of Asia, India and the Persian Gulf [30], and the strain used here was specifically selected in 1989 for susceptibility to *P. falciparum*. Recently this vector has also emerged in Africa and proven to be highly susceptible to local *P. falciparum* and *Plasmodium vivax* [31]. However, *An. stephensi* is not the dominant vector for African isolates. Previous assessments suggest that SMFA experiments with *An. stephensi* and *An. coluzzii* will not result in differences in estimated intervention efficacy [32]. However, the current studies were not designed to compare vector competence [28] that would require dedicated experiments. An additional limitation is that the current study stopped when oocysts were detected in the mosquito midgut. For a number of isolates data on sporozoite production are also available [13, 14], but comparing sporozoite production or infectivity between individual isolates was beyond the scope of this manuscript. A last limitation of the current work is that no images of non-viable gametocytes were made and expert judgement regularly informed the timing of feeding experiments. This also means that, while gametocyte-producing lines can be made available for other research purposes, gametocyte culture and SMFA remain procedures that depend on highly trained technicians and cannot always be easily transferred to other laboratories.

## Conclusions

This study reports eight *P. falciparum* clonal isolates that continuously grow in vitro, produce gametocytes, and generating reproducible oocyst numbers in *Anopheles* mosquitoes. These isolates will be of value and available to the malaria research community.

## Supplementary Information

**Additional file 1**: **Fig. S1**. Isolate infection intensity of three identical cryopreserved NF54 and NF175.D5 vials.

**Additional file 2**: **Table S1**. Geographical origin and country last visited of collected isolates. **Table S2**. Transmission results of patient isolates and derived clones to mosquito vector.

## Data Availability

The datasets used and/or analysed during the current study are available from the corresponding author on reasonable request.

## Abbreviations

MFA: Membrane feeding assay
SMFA: Standard membrane feeding assay
