# Supplementary figures and images for: A portfolio of geographically distinct laboratory-adapted Plasmodium falciparum clones with consistent infection rates in Anopheles mosquitoes
Source: Malar J. 2021 Sep 26;20:381. doi: 10.1186/s12936-021-03912-x (PMC8474906; doi:10.1186/s12936-021-03912-x)

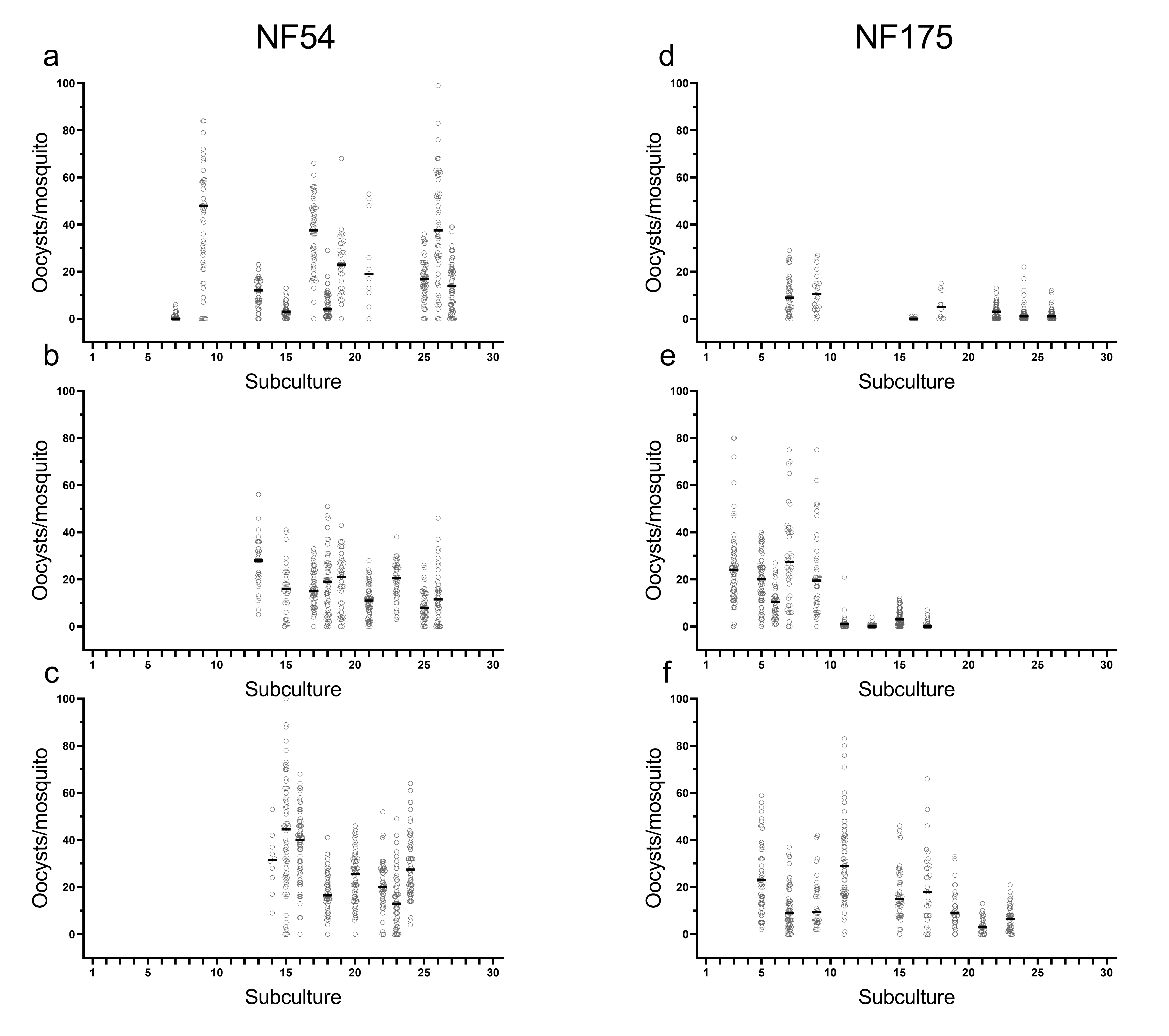

Supplement: Supplementary file 1 — Additional file 1: Fig. S1. Isolate infection intensity of three identical cryopreserved NF54 and NF175.D5 vials. [file 12936_2021_3912_MOESM1_ESM.tif]
