# Supplementary material for: A portfolio of geographically distinct laboratory-adapted Plasmodium falciparum clones with consistent infection rates in Anopheles mosquitoes
Source: Malar J. 2021 Sep 26;20:381. doi: 10.1186/s12936-021-03912-x (PMC8474906; doi:10.1186/s12936-021-03912-x)
Supplement: Supplementary file 2 — Additional file 2: Table S1. Geographical origin and country last visited of collected isolates. Table S2. Transmission results of patient isolates and derived clones to mosquito vector. [file 12936_2021_3912_MOESM2_ESM.docx]

# Additional file 2

**Table S1: Geographical origin and country last visited of collected isolates**

| **Patient isolate** | **Isolate origin (continent/region)** | **Last country last visited prior to diagnosis** |
| --- | --- | --- |
| NF28 | West Africa | Nigeria |
| NF30 | West Africa | Nigeria |
| NF33 | Central Africa | DR Congo |
| NF34 | West Africa | Nigeria |
| NF35 | Central Africa | Cameroon |
| NF38 | Origin uncertain | India* |
| NF47 | Unknown | Unknown |
| NF48 | Origin uncertain | Kenya* |
| NF50 | Southeast Asia | Indonesia |
| NF53 | West Africa | Côte d'Ivoire |
| NF54 | West Africa | Unknown |
| NF56 | East Africa | Tanzania |
| NF57 | West Africa | West Africa |
| NF58 | Southeast Asia | Indonesia |
| NF59 | West Africa | Nigeria |
| NF60 | Unknown | Unknown |
| NF61 | West Africa | Ghana |
| NF62 | Northern Africa | Mauritania |
| NF63 | East Africa | Tanzania |
| NF63R | Unknown | Unknown |
| NF64 | Unknown | Unknown |
| NF65 | East Africa | Tanzania |
| NF66 | Southeast Asia | Indonesia |
| NF67 | East Africa | Kenya |
| NF68 | Unknown | >10 years Netherlands |
| NF69 | East Africa | Tanzania |
| NF70 | Origin uncertain | Nigeria* |
| NF71 | South America | Suriname |
| NF74 | West Africa | Ghana |
| NF75 | Southeast Asia | Indonesia |
| NF81 | Unknown | Unknown |
| NF82 | Central Africa | Cameroon |

| **Patient isolate** | **Isolate origin (continent)** | **Country last visited** |
| --- | --- | --- |
| NF83 | Unknown | Unknown |
| NF84 | East Africa | Kenya |
| NF85 | East Africa | Tanzania |
| NF86 | Unknown | Unknown |
| NF87 | Central Africa | Cameroon |
| NF88 | East Africa | Kenya |
| NF89 | Unknown | Unknown |
| NF90 | East Africa | Uganda |
| NF91 | East Africa | Kenya |
| NF92 | Unknown | Unknown |
| NF93 | Unknown | Unknown |
| NF94 | Unknown | Unknown |
| NF95 | Unknown | Unknown |
| NF96 | East Africa | Tanzania |
| NF97 | Unknown | Unknown |
| NF98 | Unknown | Unknown |
| NF99 | East Africa | Sudan |
| NF100 | East Africa | Sudan |
| NF101 | Unknown | Unknown |
| NF103 | East Africa | Kenya |
| NF109 | East Africa | Kenya |
| NF110 | Unknown | Unknown |
| NF114 | Unknown | Unknown |
| NF115 | Southeast Asia | Indonesia |
| NF120 | Southern Africa | Zambia |
| NF121 | Unknown | Unknown |
| NF122 | Unknown | Unknown |
| NF123 | Unknown | Unknown |
| NF124 | Unknown | Unknown |
| NF125 | Central Africa | Cameroon |
| NF126 | East Africa | Uganda |
| NF127 | Unknown | Unknown |
| NF128 | Unknown | Unknown |
| NF129 | Unknown | Unknown |

| **Patient isolate** | **Isolate origin (continent)** | **Country last visited** |
| --- | --- | --- |
| NF130 | Unknown | Unknown |
| NF131 | Oceania | Papua New guinea |
| NF132 | Unknown | Unknown |
| NF133 | Unknown | Unknown |
| NF134 | Unknown | Unknown |
| NF135 | Southeast Asia | Cambodia |
| NF136 | West Africa | Togo |
| NF137 | Southeast Asia | Cambodia |
| NF138 | West Africa | Togo |
| NF139 | Unknown | Unknown |
| NF140 | West Africa | Niger |
| NF141 | Central Africa | Central Africa |
| NF142 | Unknown | Unknown |
| NF143 | West Africa | Senegal |
| NF144 | Origin uncertain | Indonesia* |
| NF145 | Southern Africa | South Africa |
| NF146 | Origin uncertain | Mali* |
| NF147 | West Africa | Ghana |
| NF148 | Southeast Asia | Indonesia |
| NF149 | South Asia | India |
| NF150 | West Africa | Nigeria |
| NF151 | Unknown | Unknown |
| NF152 | West Africa | Ghana |
| NF153 | South America | Frans Guyana |
| NF154 | West Africa | Nigeria |
| NF156 | South East Asia | Thailand |
| NF157 | Southeast Asia | Indonesia |
| NF158 | Southeast Asia | Indonesia |
| NF159 | East Africa | Ethiopia |
| NF162 | West Africa | Guinea |
| NF163 | West Africa | Nigeria |
| NF164 | Southeast Asia | Indonesia |
| NF165 | East Africa | Malawi |
| NF166 | West Africa | Guinea |

| **Patient isolate** | **Isolate origin (continent)** | **Country last visited** |
| --- | --- | --- |
| NF167 | West Africa | Guinea |
| NF168 | West Africa | Guinea |
| NF169 | West Africa | Guinea |
| NF170 | West Africa | Burkina Faso |
| NF171 | East Africa | Uganda |
| NF172 | East Africa | Rwanda |
| NF173 | West Africa | Ghana |
| NF174 | West Africa | Sierra Leone |
| NF175 | West Africa | Nigeria |
| NF176 | Southern Africa | Mozambique |
| NF177 | West Africa | Gambia |
| NF178 | West Africa | Gambia |
| NF179 | West Africa | Gambia |
| NF180 | East Africa | Uganda |
| NF181 | Origin uncertain | Dominican republic* |
| NF182 | Central Africa | Gabon |
| NF183 | West Africa | Togo |
| NF184 | West Africa | Burkina Faso |
| NF185 | Southern Africa | Zambia |
| NF186 | West Africa | Guinea |

Table presents geographical origin and country last visited of all collected *P. falciparum* patient isolates since 1978 at the Radboudumc and other hospitals in the Netherlands. *If unknown origin is listed but a country is mentioned, the patient traveled to multiple continents prior to diagnosis and the last country is listed but origin of the isolate was considered unknown.

**Table S2: Transmission results of patient isolates and derived clones to mosquito vector**

Table presents the membrane feeding assay (MFA) results of patient isolates and their derived clones. Infection prevalence is shown as the percentage of infected mosquitoes and infection intensity as the mean number of oocyst counted by microscopy 6-8 days post infection. MFA indicates the number of individual performed membrane feeding assays, followed by the total number of dissected mosquitoes (n).

| **Patient**  **isolate** | **Infection prevalence (%)** | **Oocyst intensity**  **(mean)** | **MFA / (n)** | **Derived**  **clone** | **Infection prevalence (%)** | **Oocyst intensity**  **(mean)** | **MFA / (n)** |
| --- | --- | --- | --- | --- | --- | --- | --- |
| NF149 | 29 | 1.1 | 7/140 | NF149.A3 | 49 | 2.8 | 27/430 |
| NF153 | 65 | 15.6 | 8/130 | NF153.E8 | 0 | 0 | 7/100 |
| NF159 | 68 | 2.4 | 4/80 | NF159.D6 | 17 | 0.3 | 4/79 |
| NF164 | 35 | 1.2 | 4/80 | NF164.C5 | 64 | 8.4 | 18/310 |
| NF165* | 25 | 0.3 | 1/20 | NF165.E4* | 64 | 14.4 | 18/310 |
| NF169 | 82 | 5 | 3/40 | NF169.E9 | 54 | 16 | 8/90 |
| NF175 | 96 | 14.3 | 12/122 | NF175.D5 | 85 | 8.6 | 21/574 |
| NF176 | 63 | 2.3 | 16/322 | NF176.B8 | 63 | 1.7 | 5/266 |
| NF183 | 87 | 12.9 | 13/212 | NF183.F7 | 73 | 8 | 7/160 |

** Transmission for this isolate was also evaluated in An. coluzzii.*

**Fig. S1 Isolate infection intensity of cryopreserved vials**

The infection intensity in oocysts per mosquito of three identical cryopreserved vials of NF54 (panel **a**, **b** and **c**) and NF175 (panel **d**, **e** and **f**). After culture initiation (zero), a subculture number was added each time fresh erythrocytes were added to the culture. Between isolates SMFAs were not always performed on the same subcultures. Bars present the mean infection intensity.
